# Integrated Hyperparameter Optimization with Dimensionality Reduction and Clustering for Radiomics: A Bootstrapped Approach

**DOI:** 10.3390/mti9050049

**Published:** 2025-05-21

**Authors:** S. J. Pawan, Matthew Muellner, Xiaomeng Lei, Mihir Desai, Bino Varghese, Vinay Duddalwar, Steven Y. Cen

**Affiliations:** 1 Radiomics Lab, University of Southern California, Los Angeles, CA 90033, USA; 2 Department of Radiology, Keck School of Medicine, University of Southern California, Los Angeles, CA 90033, USA; 3 Institute of Urology, University of Southern California, Los Angeles, CA 90033, USA; 4 Department of Radiology, Los Angeles General Medical Center, Los Angeles, CA 90033, USA; 5 Alfred E Mann Department of Biomedical Engineering, USC Viterbi School of Engineering, Los Angeles, CA 90089, USA

**Keywords:** radiomics, machine learning, clustering, hyperparameters, renal cell carcinoma

## Abstract

Radiomics involves extracting quantitative features from medical images, resulting in high-dimensional data. Unsupervised clustering has been used to discover patterns in radiomic features, potentially yielding hidden biological insights. However, its effectiveness depends on the selection of dimensionality reduction techniques, clustering methods, and hyperparameter optimization, an area with limited exploration in the literature. We present a novel bootstrapping-based hyperparameter search approach to optimize clustering efficacy, treating dimensionality reduction and clustering as a connected process chain. The hyperparameter search was guided by the Adjusted Rand Index (ARI) and Davies–Bouldin Index (DBI) within a bootstrapping framework of 100 iterations. The cluster assignments were generated through 10-fold cross-validation, and a grid search strategy was used to explore hyperparameter combinations. We evaluated ten unsupervised learning pipelines using both simulation studies and real-world radiomics data derived from multiphase CT images of renal cell carcinoma. In simulations, we found that Non-negative Matrix Factorization (NMF) and Spectral Clustering outperformed the traditional Principal Component Analysis (PCA)-based approach. The best-performing pipeline (NMF followed by K-means clustering) successfully identified all three simulated clusters, achieving a Cramér’s V of 0.9. The simulation also established a reference framework for understanding the concordance patterns among different pipelines under varying strengths of clustering effects. High concordance reflects strong clustering. In the real-world data application, we observed a moderate clustering effect, which aligned with the weak associations to clinical outcomes, as indicated by the highest AUROC of 0.63.

## 1. Introduction

Radiomics, the extraction and analysis of quantitative features from medical images, has emerged as an important and evolving area of interest in oncology research, providing valuable insights into tumor heterogeneity, treatment response, and prognosis [1–4]. The majority of radiomics literature has indeed focused on supervised learning, where models are trained to predict clinical outcomes (e.g., survival, response to treatment, tumor grade). This is because labeled outcomes are often available from clinical records, and the goal is typically to develop diagnostic or prognostic tools [5,6]. In contrast, unsupervised learning can uncover hidden patterns, intrinsic subtypes, and natural patterns. Its ability to operate without the need for ground truth makes it a valuable tool for analyzing medical data that lacks concrete, validated outcome variables. Prior studies have used combinations of PCA (Principal Component Analysis) and K-means or K-means alone for unsupervised learning [7–10]. However, there is no extended methodological study on combining dimensionality reduction with clustering methods.

Traditionally, unsupervised training pipelines have placed less emphasis on hyperparameter tuning, and cross-validation compared to supervised training. In isolated studies where hyperparameter tuning is performed, the tuning effort was limited to a single step [11]. For example, previous studies have employed PCA to maximize the total variance explained by the principal components, followed by K-means clustering, where the number of clusters was determined using the gap statistic method [12]. However, an ideal unsupervised training pipeline, which includes sequential steps such as dimensionality reduction and cluster determination, requires a cohesive strategy for hyperparameter tuning and a cross-validation procedure to prevent overfitting and ensure robustness. Notably, the literature lacks a strategy for conducting cross-validation that does not rely on ground truth.

In this study, we systematically investigate the design of different unsupervised learning methods by strategically incorporating widely used dimensionality reduction components such as t-SNE, PCA, and NMF, primarily focusing on high-dimensional radiomics data. This study is motivated by the need to capture latent features and complex relationships within the high-dimensional radiomic space by varying the methodological composition. To overcome the limitation of cross-validation without the ground truth, we propose a bootstrapping–cross-validation solution focusing on cluster reproducibility between consecutive iterations. The key principle is that successful unsupervised training should produce consistent clusters. For an individual observation, an unsupervised algorithm trained on multiple similar datasets should yield stable cluster membership with a consistent biological interpretation.

To demonstrate our method on clinical data, we employ the proposed hyperparameter optimization strategy for analyzing clear cell renal carcinoma (ccRCC). ccRCC accounts for 80% of kidney cancer cases in adults and is among the top ten most common cancers globally [13–16]. The standard diagnostic approach for ccRCC includes multiphase CT and, in select cases, biopsy. Specifically, we investigated the association between unsupervised clustering results and tumor malignancy, aggressiveness, and grading (Note: grades 1 and 2 were grouped in low-grade, and grades 3 and 4 were grouped in high-grade ccRCC [17]). To preliminarily evaluate the performance and robustness of the proposed pipelines under known conditions, we first conducted a simulation study using synthetic datasets with varying effect sizes and dimensionality. This allowed us to benchmark clustering behavior before applying the method to real-world radiomics data. To our knowledge, this study represents the first attempt to explore hyperparameter optimization through a process chain of dimensionality reduction and cross-validation modules in unsupervised learning.

## 2. Materials and Methods

This section provides a comprehensive overview of the datasets used, including both clinical and simulated data, followed by the methodological framework involving unsupervised learning pipelines and hyperparameter tuning.

### 2.1. Datasets

#### 2.1.1. Clinical Data

##### Patient Demographics

This IRB-approved, HIPAA-compliant study included 447 patients with ccRCC who underwent nephrectomy at USC between 2009 and 2018, with pre-nephrectomy multiphase clinical CT data available. The cohort consisted of 305 male patients (68.2%) and 142 female patients (31.8%), with an average age of 60.7 ± 12.6 years (IQR: 52–70). Of the total cases, 24.6% were benign, and 75.4% were malignant. Among the malignant cases, 68.4% were grade 1 or 2 RCC, while 31.6% were grade 3 or 4. Additionally, 62.2% of the malignant tumors were classified as non-aggressive and 37.8% as aggressive (Table 1).

##### Preprocessing and Data Extraction

For both training and validation sets, a 13-parameter linear 3D co-registration was completed using the Statistical Parametric Mapping (SPM) software package (version 12) (Welcome Trust Centre for Neuroimaging) in MATLAB^®^ (MathWorks, Natick, MA, USA) [18]. Radiomic features were extracted using Pyradiomics [19], an open-source tool for high-throughput extraction of quantitative imaging biomarkers. These features capture tumor heterogeneity by analyzing spatial intensity distributions, texture patterns, and shape descriptors. These features encompass multiple families, including shape-based, first-order statistical, and higher-order texture features. Shape-based features describe the geometric properties of the tumor, while first-order statistics quantify intensity distribution within the region of interest. Higher-order texture features are derived from Gray Level Co-occurrence Matrix (GLCM), Gray Level Size Zone Matrix (GLSZM), Gray Level Run Length Matrix (GLRLM), Gray Level Dependence Matrix (GLDM), and Neighboring Gray Tone Difference Matrix (NGTDM), capturing spatial relationships and patterns within the image. These diverse feature families provide a comprehensive characterization of tumor heterogeneity.

#### 2.1.2. Simulated Data

To evaluate and compare pipeline performance, we simulated data using a mixture of K multivariate normal distributions. The simulation parameters were derived from Fisher’s Iris dataset, which provides a ground truth of three iris species. We generated three levels of cluster separation: (1) small, with approximately 10% differences in means, (2) medium, with approximately 20% differences, and (3) large, with approximately 50% differences. Each set of simulated data contained 1000 individual observations, and each level of cluster separation included sets with 0, 4, 20, 40, 60, 80, 140, and 200 covariates beside the four features that were used to construct the ground truth. The simulated datasets underwent the same preprocessing steps as the clinical data to ensure consistency in pipeline evaluation.

Cluster labels were recorded for each observation and compared with the ground truth labels, generating a Cramér’s V score, which quantified the association between the model-identified clusters and the simulated ground truth without requiring the match of cluster number. This scoring metric is useful when clusters may be equal across samples, while cluster labels differ.

### 2.2. Methods

#### 2.2.1. Unsupervised Learning Pipelines

**Figure 1.**
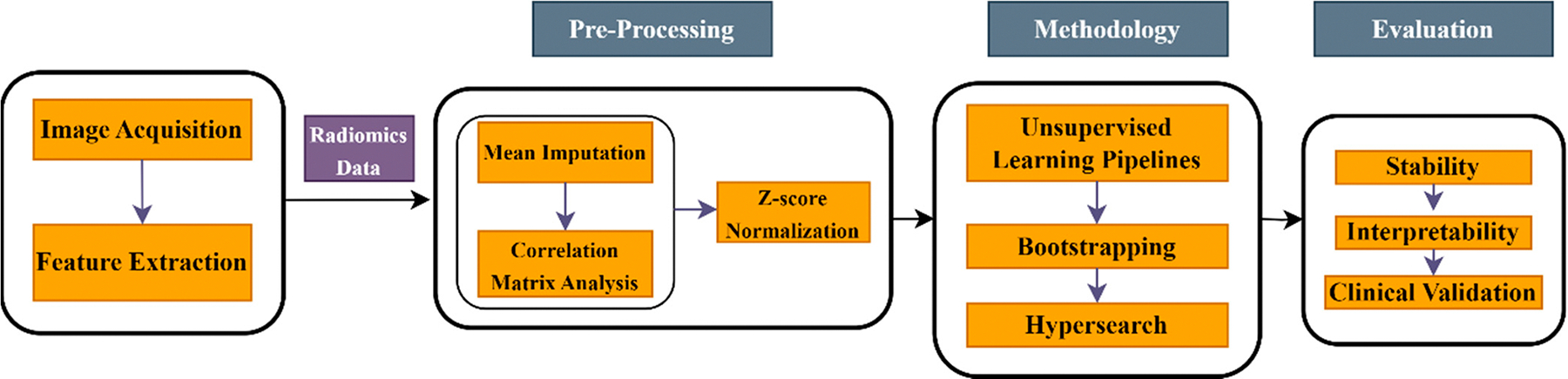
Represents the workflow of the proposed method. Image acquisition and feature extraction are performed in stage 1, followed by subjecting radiomics data to pre-processing (stage 2) involving a mean imputer, correlation matrix analysis, and z-score normalization. We perform a hyperparameter search with bootstrapping (stage 3) on different unsupervised clustering pipelines with chain dimensionality reduction modules. Finally, in stage 4, we evaluate the performance for stability, interpretability, and clinical validation.

This manuscript presents a novel bootstrap-based method for hyperparameter search. Figure 1 depicts the workflow followed in various unsupervised learning pipelines in this project. The procedure is guided by the ARI and DBI, evaluated through 10-fold cross-validation. Using a grid-search strategy, hyperparameter combinations were determined from bootstrapped samples (90% sampled with replacement). The ARI of each consecutive pair of bootstrapped samples was generated, and the final value was computed as the average across 100–1 pairwise comparisons. The final DBI value was the mean DBI across the bootstrapped samples. Table 2 presents the list of hyperparameters considered across different pipeline components. Ten different pipelines were implemented, as listed in Table 3. The first pipeline (M1) applied K-means directly to the radiomics data without dimensionality reduction, serving as a baseline to assess the necessity of dimensionality reduction for high-dimensional datasets. The second pipeline (M2) incorporated PCA before K-means to capture essential variations. The third pipeline (M3) combined NMF and PCA for dimensionality reduction before applying K-means, while the fourth pipeline (M4) applied only NMF before K-means. The fifth pipeline (M5) used Spectral clustering without prior dimensionality reduction, whereas the sixth (M6) included PCA before Spectral clustering. The seventh pipeline (M7) followed a sequence of NMF, PCA, and Spectral clustering, while the eighth (M8) applied NMF before Spectral clustering. The ninth pipeline (M9) integrated t-SNE (t-distributed Stochastic Neighbor Embedding) after PCA before K-means, and the tenth (M10) used t-SNE directly before K-means clustering. All machine learning experiments were conducted using Python’s scikit-learn library.

To ensure the reproducibility of the model, we have implemented a 10-fold cross-validation; thus, the cluster membership used for the model assessment was based on the result from the 10 mutually exclusive testing samples. For Spectral clustering, since the sklearn library cannot save the constructed model from the training sample, we have created a surrogate procedure using supervised learning to “record” the memory of the clustering algorithm in the training phase. A k nearest neighbor procedure was used to score the independent testing data and obtain the cluster membership.

#### 2.2.2. Hyperparameter Tuning

Hyperparameter optimization was performed using a grid-search strategy with bootstrapping and 10-fold cross-validation, as described earlier. The top five hyperparameter combinations were selected based on the highest mean ARI. ARI measures clustering stability by quantifying the similarity between two data partitions across bootstrapped samples. This metric was chosen to ensure that the selected hyperparameter combination produced the most reproducible results. From this set of five, the final hyperparameter configuration for each model (e.g., NMF + K-means) was determined based on the lowest mean Davies–Bouldin Index (DBI) score across all bootstrapped samples. The Davies–Bouldin Index (DBI) measures cluster compactness, with lower scores indicating tighter intra-cluster grouping and greater separation between distinct clusters. DBI helps prevent oversimplification in cluster selection, as relying solely on ARI tends to favor fewer clusters (often just two) to maximize stability. Incorporating DBI into the decision rule counterbalances this tendency, ensuring a more balanced cluster selection. The cutoff for ARI-based ranking can be relaxed, for example, selecting the top 10 combinations, to place greater emphasis on DBI. Additionally, the clinical interpretability of the resulting clusters can serve as an important factor in guiding the final selection.

This way, cluster 1 in the ground truth can be allowed to match with cluster 2 in the pipeline. The best method would identify three clusters at high numbers of covariates and achieve the highest Cramér’s V (close to 1) among peer models.

#### 2.2.3. Performance Assessment

We assessed performance using three criteria: (1) cluster robustness, (2) cluster interpretability, and (3) the clinical relevance of clusters derived from unsupervised training. For hyperparameter selection, we used the ARI and Davies–Bouldin Index (DBI) as the key metrics to identify the optimal hyperparameter. ARI also served as a benchmark for comparing model robustness, where a robust model exhibited a higher average ARI across bootstrapping iterations. To assess cluster interpretability, we employed a Classification Tree (CART^®^) to “unpack” the clusters, which condensed high-dimensional radiomic data. CART provides transparency by revealing the predictor combinations that define each cluster. We further examined the biological significance of these radiomic interpretations. The accuracy of the CART was evaluated using 10-fold cross-validation. The clinical relevance of the clusters was determined by their association with malignancy, aggressiveness, and tumor grade, which were assessed using logistic regression and quantified by AUROC. To examine cluster similarity across the 10 models, we computed Cramér’s V for all model pairs. For statistical analysis, we used Salford Predictive Modeler 8 for CART^®^ and SAS 9.4 for all other assessments.

## 3. Results

### 3.1. Simulation Study

We assessed the concordance between cluster membership and iris species categories. Strong alignment of cluster membership with iris species would denote good unsupervised learning pipeline performance. The best performance was defined by two criteria: (1) detecting three clusters and (2) achieving the highest Cramér’s V. Overall, we observed that agreement with the ground truth decreased as effect size diminished. Additionally, as the number of covariates increased, agreement further declined. The t-SNE->PCA->K-means (M9) and t-SNE->K-means (M10) pipelines consistently performed poorly, regardless of effect size or the number of confounders (Supplementary Table S1). We also noted that agreement between pipelines was stronger when the effect size was large, meaning models were more likely to produce consistent clusters. With a medium effect size, pipelines within the same family still showed good agreement. For example, the Cramér’s V values between NMF-Spectral (M8) and NMF->PCA->K-means (M3), NMF->K-means (M4), and Spectral (M5) were 0.71, 0.49, and 0.78, respectively. However, when the effect size was small, these agreements dropped significantly to 0.08, 0.30, and 0.12 (Supplementary Table S2).

**Figure 2.**
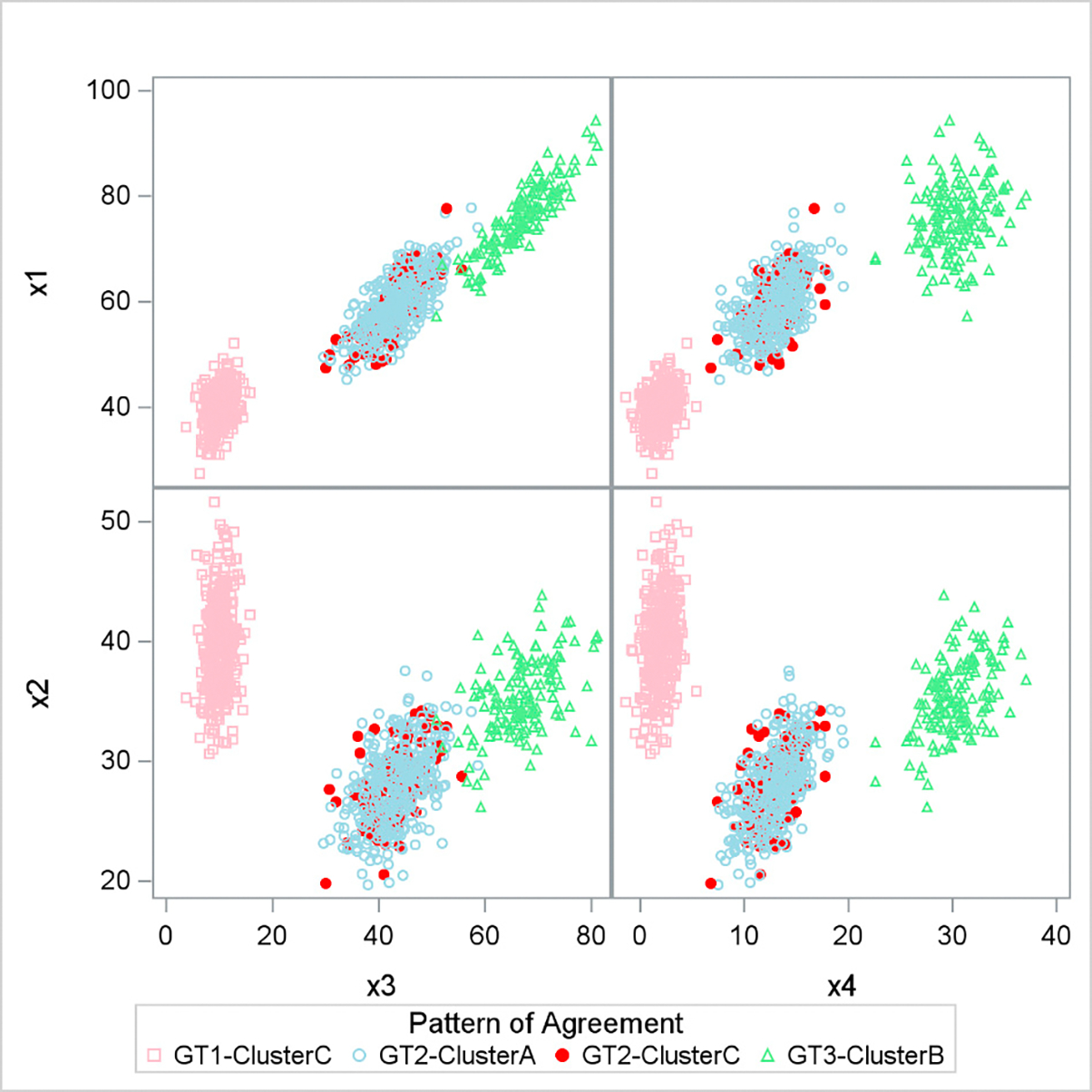
Overlap between cluster detected and iris species observed. Legend: GT1-GT3: Ground truths 1–3 represent the three iris species; clusters A-C are the clusters detected by our unsupervised learning pipeline. Red circle filled: Cluster C mismatched with GT2. X1–X4: Features used to simulate iris species, which represented different unique aspects of different species.

Our results showed that the NMF->K-means (M4) pipeline with 200 covariates in large effect size achieved the best performance, capturing all 3 clusters with Cramér’s V = 0.9 (Supplementary Table S1). Figure 2 illustrates the overlap between the three clusters and the actual iris species. It is apparent that cluster C perfectly matched iris species 1, while cluster B aligned entirely with iris species 3. Most cases in cluster A corresponded to iris species 2, though some instances from cluster C were also mixed into this category.

### 3.2. Real-World Data Application

The real-world data results followed a similar pattern to the simulated data with a medium effect size. The agreement between unsupervised clusters and clinical outcomes was relatively weak, with Cramér’s V ranging from 0.05 to 0.45 (Supplementary Table S3). Agreement across different pipelines varied between 0.13 and 0.66, while agreement within the same pipeline family remained relatively strong. For example, the Cramér’s V values between NMF->Spectral (M8) and NMF->PCA->K-means (M3), NMF->K-means (M4), and Spectral were 0.53, 0.42, and 0.35, respectively falling between the findings from medium and small effect sizes in the simulation study. Similarly, the agreement with clinical metrics was consistent with the strength levels observed for small to medium effect sizes in the simulated data. None of the pipelines showed strong alignment with clinical metrics, with AUROC values not exceeding 0.63.

Regarding stability, NMF->PCA->K-means (M3), NMF->K-means (M4), and NMF->PCA->Spectral (M7) demonstrated outstanding repeatability, achieving ARI scores of 0.83, 0.82, and 0.82, respectively (Table 4). In contrast, t-SNE->PCA->K-means and t-SNE->K-means were too unstable to produce an ARI score. Our results also showed that NMF->PCA->Spectral and NMF->Spectral had the best interpretability, with an overall 10-fold cross-validation accuracy of 69.57% and 69.35%, respectively. In comparison, K-means, t-SNE->PCA->K-means, and t-SNE->K-means had the lowest interpretability, with overall predictive accuracies of 43.62%, 57.05%, and 30.20%, respectively.

## 4. Discussion

This study presents a novel approach to unsupervised learning by conducting hyperparameter tuning across multiple computing steps in a unified framework. Unlike prior studies that perform tuning in isolated steps, our method integrates dimensionality reduction, clustering, and hyperparameter selection into a cohesive process, optimizing the entire pipeline. To ensure model reproducibility, we implemented a 10-fold cross-validation strategy alongside bootstrapping. This approach allowed us to assess the stability of clustering results across different iterations, mitigating overfitting and enhancing robustness. Additionally, we employed a bootstrapping-based strategy to derive both the Adjusted Rand Index (ARI) and Davies–Bouldin Index (DBI), ensuring a balanced evaluation of clustering performance.

Our study further introduced a rigorous performance evaluation framework. We assessed model performance on both simulated and real-world radiomics data, allowing systematic comparison under controlled conditions and clinical relevance. Performance was evaluated based on three key criteria: (1) cluster robustness, which is measured through ARI across bootstrapped iterations; (2) cluster interpretability, which is assessed using a classification tree (CART) to unpack high-dimensional radiomic features; and (3) clinical relevance, which is determined by the association of clusters with malignancy, aggressiveness, and tumor risk using logistic regression and AUROC. Additionally, intermodel agreement was quantified using Cramér’s V to assess clustering consistency across different pipelines. Please refer to the Supplementary Material for CART analysis.

The true strength of clustering structure remains unknown when using highdimensional imaging data in cancer research. Pathological subtypes may not fully account for the observed variation in radiological image expression [20]. Studying the simulation data allowed us to evaluate the model performance under different scenarios, from large to small clustering effect. By generating datasets with varying degrees of cluster separation, we tested how well each pipeline could recover the underlying structures. The results demonstrated that the clustering performance degraded as the effect size decreased and the number of covariates increased. This simulation-based evaluation provided critical insights into the robustness of different dimensionality reduction and clustering approaches, offering a controlled framework for validating real-world applicability. One notable observation in our study was the inconsistency in clustering results when different dimensionality reduction techniques were applied. Pipelines utilizing PCA, Spectral, and NMF showed varying degrees of agreement, particularly when the effect size was small. The observed disagreement suggests that different dimensionality reduction techniques capture distinct latent structures, influencing the clustering outcome. This variability underscores the importance of selecting an appropriate dimensionality reduction method tailored to the dataset’s characteristics. However, when dimensionality reduction was overapplied, such as combining PCA with the Spectral clustering method, the performance declined significantly (Supplementary Table S1).

Among the dimensionality reduction techniques evaluated, NMF consistently demonstrated superior cluster stability. NMF also enhanced cluster interpretability, especially in combination with Spectral clustering. NMF is a non-negative matrix factorization technique that decomposes data into additive components. Unlike PCA, which projects data onto orthogonal axes, NMF preserves the parts-based representation of the data, making it more interpretable in identifying biologically meaningful patterns. The superior performance of NMF in our study suggests that it effectively captures the latent structure of radiomic features, making it a strong candidate for dimensionality reduction in unsupervised radiomics analysis. Our findings also highlight the instability of t-SNE when used as a dimensionality reduction step before clustering. Unlike linear techniques such as PCA and NMF, t-SNE is a non-linear embedding method designed for visualizing high-dimensional data rather than preserving global structure [21]. Since t-SNE is sensitive to initialization and parameter choices (e.g., perplexity), its output can vary significantly across runs, leading to inconsistent clustering results. Furthermore, since the scikit-learn implementation does not retain the learned transformation, applying t-SNE separately to training and testing datasets can result in inconsistent mappings, further contributing to instability [12,16]. These limitations explain why pipelines incorporating t-SNE (M9 and M10: Supplementary Table S1) performed poorly in our analysis.

One key observation in our real-life data application was the relatively poor agreement between unsupervised clusters and clinical metrics, as reflected by the low to moderate Cramér’s V values. This finding aligns with existing literature [22], which has reported no statistical association between radiomics-based clustering and clinical outcomes, including histological grade (chi-square = 0.2316, *p* = 0.8907) and clinical stage (chi-square = 6.3583, *p* = 0.3843). Several factors may contribute to this discrepancy. First, radiomics features capture high-dimensional, complex representations of tumor heterogeneity that may not always align with traditional clinical categorizations, such as malignancy or tumor aggressiveness. Second, clinical metrics are often derived from histopathological assessments, which may not fully reflect the continuous spectrum of biological variations captured by radiomics. Prior studies have shown that while radiomics features can provide valuable prognostic information, their direct correspondence to clinical endpoints remains inconsistent due to factors such as interobserver variability in annotations, imaging acquisition differences, and the inherent complexity of tumor biology [23–25]. These findings highlight the need for further refinement in linking radiomics-derived clusters with meaningful clinical interpretations.

Our study offers a reference for interpreting the underlying effect size of latent clusters using simulated data. For instance, when a medium effect size is present in the simulated dataset, the majority of Cramér’s V values fall between 0.3 and 0.7, indicating moderate agreement across different clustering pipelines. In scenarios where ground truth labels are unavailable, this inter-pipeline agreement can serve as an indirect measure of the effect size of latent clusters. In other words, if most of the Cramér’s V values across pipelines lie within the 0.3–0.7 range, it suggests sufficient separation among clusters to justify further exploration. Conversely, if most of the Cramér’s V values fall below 0.3, it may indicate a weak clustering structure, discouraging further investigation. In our clinical application, we observed Cramér’s V values consistent with a medium effect size, supporting continued exploration of our clustering results.

Given the variability in clustering outcomes and the observed disagreement across different dimensionality reduction methods, we recommend a multi-pipeline approach for future studies. By applying multiple unsupervised pipelines and assessing their output consistency, researchers can identify the most suitable method for their specific data characteristics. This approach would allow for a more robust estimation of clustering reliability, reducing the impact of biases introduced by any single dimensionality reduction or clustering technique. Additionally, our study demonstrates that hyperparameter tuning and cross-validation play a crucial role in preventing false discoveries. Incorrectly identifying clusters when the true biological cluster does not exist can mislead conclusions and waste clinical resources. This issue is particularly critical in radiomics, where overfitting due to high-dimensional data can generate spurious associations. The literature suggests that false discoveries in high-dimensional data are more problematic than null findings, as they can lead to unreliable clinical decision-making [26]. By implementing robust hyperparameter selection and cross-validation, we mitigate the risk of overfitting, ensuring that detected clusters are reproducible and biologically meaningful rather than artifacts of data noise or methodological bias.

## 5. Conclusions

This study presents a comprehensive framework for unsupervised learning in highdimensional radiomics data, integrating dimensionality reduction, clustering, and hyperparameter optimization within a unified, bootstrapping-based pipeline. Through both simulation and real-world ccRCC datasets, we demonstrated that clustering performance is highly sensitive to the effect size; as effect size diminished, agreement with ground truth declined, and this effect was further exacerbated by the presence of confounding variables. Our findings also reveal that t-SNE-based pipelines were prone to inconsistency and poor reproducibility. The relatively weak alignment between radiomics-derived clusters and clinical metrics highlights the complexity of mapping high-dimensional imaging features to traditional clinical labels. These insights emphasize the importance of robust hyperparameter tuning, multi-pipeline evaluation, and effect size considerations to ensure the reproducibility, interpretability, and translational value of unsupervised radiomics analysis.

## Supplementary Material

Supplementary data

**Supplementary Materials:** The following supporting information can be downloaded at: https://www.mdpi.com/article/10.3390/mti9050049/s1, Table S1: Agreement (Cramér’s V) with Ground Truth for Each Pipelines from Simulated Data; Table S2: Agreement (Cramér’s V) across Pipelines in Simulated Data with 200 Covariates by Effect Size; Table S3: Agreement across Pipelines from Real-World Data Application.

## Figures and Tables

**Table 1.** Demographic details of patients with Clear Cell Renal Cell Carcinoma (ccRCC).

ccRCC Demographics		
Characteristics		Sample Size
Gender	Male	305 (68.2%)
	Female	142 (31.8%)
Age		Mean 60.7 ± 12.6, Median 62 (52 to 70)
Grade		
• Benign		110 (24.6%)
• Malignant		337 (75.4%)
• Low (1 or 2)		223 (68.4%)
• High (3 or 4)		103 (31.6%)
• Non-Aggressive		278 (62.2%)
• Aggressive		169 (37.8%)

**Table 2.** Hyperparameters of the various sub-modules adopted in the unsupervised pipelines.

Method	Hyperparameters
K-means	Number of clusters
Kernel PCA	Kernels 1. Sigmoid 2. RBF 3. Linear 4. Cosine 5. Polynomial PCA components
t-SNE	Perplexity
NMF	NMF-Components
Spectral	Number of clusters

**Table 3.** Unsupervised learning pipelines used in analysis of clinical data.

Pipelines	Pipelines
M1	K-means
M2	PCA->K-means
M3	NMF-> PCA ->K means
M4	NMF ->K- means
M5	Spectral
M6	PCA-> Spectral
M7	NMF-> PCA ->Spectral
M8	NMF-> Spectral
M9	t-SNE-> PCA ->K-means
M10	t-SNE-> K-means

**Table 4.** Cluster robustness, interpretability, and association with clinical metrics from highdimensional ccRCC radiomics data.

	Robustness	Interpretability	Aggressive	Malignant	Risk 1 vs. 23	Risk 2 vs. 13	Risk 3 vs. 12
K-means	0.43	43.62%	0.6 95% CI (0.54, 0.65)	0.59 95% CI (0.53, 0.65)	0.57 95% CI (0.51, 0.63)	0.56 95% CI (0.5, 0.63)	0.58 95% CI (0.49, 0.66)
PCA-K-means	0.65	68.46%	0.59 95% CI (0.54, 0.64)	0.61 95% CI (0.55, 0.67)	0.54 95% CI (0.49, 0.6)	0.51 95% CI (0.45, 0.58)	0.57 95% CI (0.49, 0.64)
NMF-PCA-K-means	0.83	62.42%	0.56 95% CI (0.51, 0.61)	0.57 95% CI (0.52, 0.63)	0.57 95% CI (0.52, 0.62)	0.55 95% CI (0.48, 0.61)	0.56 95% CI (0.49, 0.63)
NMF-K-means	0.82	64.88%	0.59 95% CI (0.53, 0.64)	0.56 95% CI (0.5, 0.61)	0.59 95% CI (0.54, 0.64)	0.57 95% CI (0.51, 0.64)	0.57 95% CI (0.49, 0.64)
Spectral	0.36	Not able to produce	0.58 95% CI (0.52, 0.63)	0.63 95% CI (0.58, 0.69)	0.53 95% CI (0.48, 0.59)	0.51 95% CI (0.44, 0.57)	0.52 95% CI (0.44, 0.61)
PCA-Spectral	0.65	51.01%	0.5 95% CI (0.45, 0.55)	0.52 95% CI (0.46, 0.58)	0.54 95% CI (0.49, 0.6)	0.59 95% CI (0.52, 0.65)	0.52 95% CI (0.45, 0.6)
NMF-PCA-Spectral	0.82	69.57%	0.54 95% CI (0.49, 0.6)	0.54 95% CI (0.49, 0.6)	0.51 95% CI (0.45, 0.56)	0.5 95% CI (0.44, 0.57)	0.51 95% CI (0.43, 0.59)
NMF-Spectral	0.7	69.35%	0.59 95% CI (0.54, 0.64)	0.62 95% CI (0.57, 0.68)	0.54 95% CI (0.49, 0.59)	0.51 95% CI (0.45, 0.57)	0.54 95% CI (0.46, 0.63)
t-SNE-PCA-K-means	Not stable	57.05%	0.56 95% CI (0.51, 0.61)	0.57 95% CI (0.51, 0.62)	0.53 95% CI (0.48, 0.58)	0.53 95% CI (0.47, 0.58)	0.51 95% CI (0.44, 0.59)
t-SNE-K-means	Not stable	30.20%	0.53 95% CI (0.48, 0.58)	0.55 95% CI (0.49, 0.61)	0.5 95% CI (0.45, 0.55)	0.53 95% CI (0.46, 0.59)	0.53 95% CI (0.46, 0.6)

Note: Robustness captures how consistently a pipeline assigns clusters across bootstrap samples, quantified by the mean ARI score. Interpretability refers to the likelihood that a radiomics profile can be constructed to represent the cluster membership. This metric is given by the overall prediction accuracy of a classification tree constructed using the cluster membership.

## Data Availability

The data presented in this study are available from the corresponding author upon request, subject to ethical and institutional approvals. Data generated or analyzed during the study are available from the corresponding author by request and institutional review. Deidentified data may be available per university IRB rules and regulations for private review access.
