# Supplementary material for: Integrated Hyperparameter Optimization with Dimensionality Reduction and Clustering for Radiomics: A Bootstrapped Approach
Source: Multimodal Technol Interact. Author manuscript; Available in PMC 2026 Mar 27. (PMC13020691; doi:10.3390/mti9050049)
Supplement: Supplementary data [file NIHMS2148722-supplement-Supplementary_data.pdf]

Supplementary Table S1: Agreement (Cramér's V) with Ground Truth for Each Pipelines from Simulated Data

Effect Size	Cov Num	K-means	PCA-> K-means	NMF->PCA ->K-means	NMF->K-means	Spectral	PCA-> Spectral	NMF->PCA -> Spectral	NMF-> Spectral	t-SNE->PCA-> KMeans	t-SNE-> KMeans
large	0	0.2	1	0.5	0.2	1	0.6	0.3*	0.3*	0.2*	0
large	4	0.2	1	0.2	0.5*	0.8	0.7	0.2	0.3	0.1	4
large	20	1	0.8	0.2*	0.4*	1	0.6	0.2	0.3	0.2	20
large	40	0.2	1	0.2	0.6	1	0.2	0.3	0.3	0.1	40
large	60	1	1	1	0.7	1	0.5	0.3	0.4	0.4	60
large	80	0.4	1	0.8	1	1	0.5	0.3*	0.8	0.2	80
large	140	0.6	1	1	0.8*	1	0.1*	0.1*	0.6*	0.5	140
large	200	1	0.5	0.8	0.9*	1	0.1	0.1	1	0.6	200
medium	0	0.2	1	0.3	0.3	0.8	0.3	0.7	0.5	0.2*	0
medium	4	1	1	0.5	0.4	1	0.2	0.2	0.1	0.3	4
medium	20	0.2	0.6	0.1	0.3	0.8	0.6	0.4	0.2	0.2	20
medium	40	1	0.4	0.4	0.3	0.9	0.4	0.3	0.2	0.2	40
medium	60	0.6	0.4	0.4	0.6	0.5	0.7	0.4	0.2	0.2	60
medium	80	1	0.3	0.3	0.3	0.6	0.5	0.2	0.3	0.4	80
medium	140	0.4	0.4	0.8	0.6	0.6	0.5	0.2	0.8	0.1	140
medium	200	0.4	0.3	1	0.7	0.6	0.3	0.4	0.7	0.2	200
small	0	0.1	0.7	0.3	0.3	0.7	0.5	0.3	0.3	0.2*	0
small	4	0.1	1	0.2	0.2*	0.7	0.5	0.1*	0.4	0.1	4
small	20	0.1	0.3	0.1	0.1*	0.6	0.2	0.2	0.1	0.1	20
small	40	0.2	0.6	0.2	0.3	0.3*	0.3	0.1	0.2	0.1	40
small	60	0.3	0.5	0.1	0	0.3*	0.2	0.2*	0.1	0.2	60
small	80	0.2	0.5	0.1	0.4	0.3*	0.3	0.2	0.2	0.2	80
small	140	0	0.3	0.5	0.4	0.3*	0.2	0.1	0	0.2	140
small	200	0.1	0.5	0.8	0.2	0.3*	0.2	0.2	0.3	0.2	200

Note: * Model produced 3 clusters

Supplementary Table S2: Agreement (Cramér's V) across Pipelines in Simulated Data with 200 Covariates by Effect Size

Effect Size	pipeline1	K-means	PCA-> K-means	NMF->PCA-> K-means	NMF->K-means	Spectral	PCA-> Spectral	NMF-> PCA -> Spectral	NMF -> Spectral	t-SNE->PCA-> K-means
large	PCA->K-means	0.54								
large	NMF->PCA->K-means	0.78	0.79							
large	NMF->K-means	0.81	0.41	0.60						
large	Spectral	0.99	0.54	0.78	0.81					
large	PCA->Spectral	0.14	0.17	0.18	0.12	0.14				
large	NMF->PCA->Spectral	0.13	0.13	0.19	0.03	0.12	0.17			
large	NMF->Spectral	1.00	0.54	0.78	0.81	0.99	0.14	0.13		
large	t-SNE->PCA->K-means	0.64	0.55	0.67	0.47	0.64	0.11	0.06	0.64	
large	t-SNE->K-means	0.02	0.36	0.25	0.05	0.03	0.10	0.08	0.02	0.15
medium	PCA->K-means	-0.01								
medium	NMF->PCA->K-means	0.37	-0.17							
medium	NMF->K-means	0.27	-0.11	0.72						
medium	Spectral	0.29	0.45	0.78	0.65					
medium	PCA->Spectral	-0.12	0.24	-0.22	-0.04	0.39				
medium	NMF->PCA->Spectral	0.02	-0.02	0.10	0.33	0.42	0.19			
medium	NMF->Spectral	0.25	0.27	0.71	0.49	0.78	0.03	0.18		
medium	t-SNE->PCA->K-means	0.11	0.24	0.22	0.36	0.48	0.14	0.23	0.37	
medium	t-SNE->K-means	0.07	-0.11	0.22	0.17	0.21	-0.10	-0.01	0.04	-0.01
small	PCA->K-means	-0.01								
small	NMF->PCA->K-means	-0.14	-0.30							
small	NMF->K-means	-0.04	0.01	0.35						
small	Spectral	0.06	0.22	0.20	0.17					
small	PCA->Spectral	0.09	0.12	0.18	0.15	0.11				
small	NMF->PCA->Spectral	-0.04	-0.13	0.10	0.12	0.03	0.11			
small	NMF->Spectral	-0.09	0.06	0.08	0.30	0.12	0.12	0.10		
small	t-SNE->PCA->K-means	-0.05	-0.01	0.19	0.06	0.05	0.14	0.02	-0.18	
small	t-SNE->K-means	0.03	0.09	0.08	0.08	0.12	0.07	0.07	0.03	0.06

Supplementary Table S3: Agreement across Pipelines from Real-World Data Application

	K-means	PCA ->K-means	NMF ->PCA-> K-means	NMF-> K- means	Spectral	PCA-> Spectral	NMF ->PCA-> Spectral	NMF-> Spectral	t-SNE-> PCA-> K-means	t-SNE-> K-means	Malignant	Aggressive
PCA->K-means	0.51											
NMF PCA K-means	0.42	0.34										
NMF K-means	0.46	0.22	0.66									
Spectral	0.32	0.34	0.31	0.25								
PCA Spectral	0.34	0.34	0.31	0.26	0.25							
NMF PCA Spectral	0.4	0.34	0.41	0.38	0.39	0.33						
NMF Spectral	0.38	0.36	0.53	0.42	0.35	0.37	0.72					
t-SNE PCA K-means	0.22	0.18	0.18	0.13	0.45	0.2	0.25	0.34				
t-SNE K-means	0.25	0.23	0.34	0.31	0.17	0.28	0.22	0.24	0.25			
Malignant	0.29	0.22	0.15	0.09	0.35	0.12	0.2	0.21	0.12	0.16		
Aggressive	0.27	0.17	0.11	0.16	0.23	0.07	0.2	0.17	0.11	0.07	0.45	
Risk	0.15	0.08	0.11	0.11	0.12	0.1	0.05	0.07	0.05	0.12	0.39	0.38
